# Antimicrobial activity of ceftazidime-avibactam against KPC-2-producing Enterobacterales: a cross-combination and dose-escalation titration study with relebactam and vaborbactam

**DOI:** 10.1128/spectrum.00344-24

**Published:** 2024-04-30

**Authors:** Min Seo Kang, Jin Yang Baek, Jae-Hoon Ko, Sun Young Cho, Keon Young Lee, Young Ho Lee, Jinyoung Yang, Tae Yeul Kim, Hee Jae Huh, Nam Yong Lee, Kyungmin Huh, Cheol-In Kang, Doo Ryeon Chung, Kyong Ran Peck

**Affiliations:** 1 Division of Infectious Diseases, Department of Medicine, Samsung Medical Center, Sungkyunkwan University School of Medicine, Seoul, South Korea; 2 Asia Pacific Foundation for Infectious Diseases (APFID), Seoul, South Korea; 3 Centre for Infection Prevention and Control, Samsung Medical Center, Seoul, South Korea; 4 Department of Laboratory Medicine and Genetics, Samsung Medical Center, Sungkyunkwan University School of Medicine, Seoul, South Korea; Duke University, Durham, North Carolina, USA

**Keywords:** KPC, avibactam, relebactam, vaborbactam, susceptibility

## Abstract

With the introduction of ceftazidime-avibactam worldwide, the antimicrobial activity of new β-lactam/β-lactamase inhibitors (BL/BLIs) needs to be investigated. From January 2020 to June 2023, *Klebsiella pneumoniae* carbapenemase (KPC)-producing Enterobacterales were collected. With a broth microdilution test of new BL/BLIs, cross-activity test with nine combinations of BLs and new BLIs and dose-escalation titration test for non-susceptible isolates were conducted to investigate inhibitory activities of new BLIs. A total of 188 isolates was collected and most isolates (186/188, 98.9%) carried the KPC-2 gene exclusively, while two isolates (1.1%) co-harbored NDM-1. Among the 186 KPC-2-producing isolates, 184 (98.9%) were susceptible to ceftazidime-avibactam, 173 (93.0%) to imipenem-relebactam, and 184 (98.9%) to meropenem-vaborbactam. All isolates non-susceptible to imipenem-relebactam or meropenem-vaborbactam became susceptible when avibactam replaced relebactam or vaborbactam, with 7 of 11 (63.6%) imipenem-relebactam non-susceptible isolates and both (100.0%) of the meropenem-vaborbactam non-susceptible isolates. When the minimum inhibitory concentrations (MICs) of BLs were compared using log_2_ scales, combinations with avibactam showed statistically significant efficacy in lowering MICs compared to relebactam and vaborbactam (all *P* < 0.05). In the dose-escalation test of new BLIs, increasing dose of all new BLIs corresponded to increased susceptibility to BLs. Ceftazidime-avibactam exhibited excellent susceptibility against KPC-2-producing Enterobacterales unless co-harboring metallo-β-lactamase. The cross-combination test against non-susceptible isolates suggests that the inhibitory activity of avibactam was superior to those of relebactam or vaborbactam. Increasing the dose of new BLIs produced increased susceptibility to BLs, suggesting that high-concentration regimen need to be developed.

**IMPORTANCE:**

This study investigated 188 *Klebsiella pneumoniae* carbapenemase (KPC)-2-producing Enterobacterales collected from January 2020 to June 2023 in a tertiary care hospital of Korea. Most isolates were susceptible to ceftazidime-avibactam (98.9%) and meropenem-vaborbactam (98.9%), while susceptibility to imipenem-relebactam was lower (93.0%). The cross-combination test using nine combinations of the individual β-lactams (BLs) and new β-lactamase inhibitors (BLIs) showed that the inhibitory activity of avibactam was significantly superior to relebactam or vaborbactam when the Log_2_ MIC of BLs were compared for each combination with BLIs (all *P* < 0.05). The dose-escalation test of new BLIs demonstrated that increasing doses of new BLIs corresponded to increased susceptibility to BLs. Taken together, this study illustrates the excellent activity of ceftazidime-avibactam against KPC-2-producing Enterobacterales and suggests further investigation into high-concentration regimens for potentially non-susceptible clinical isolates.

## INTRODUCTION

The rapidly increasing prevalence of carbapenem-resistant Enterobacterales (CRE) is emerging as a serious global health concern, primarily due to limited treatment options, high mortality rates, and a substantial economic burden (1–3). Carbapenemase-producing Enterobacterales (CPE) play a crucial role in the spread of CRE through transfer of carbapenemase-harboring plasmids and clonal dissemination (4–6). Recent CPE outbreaks have been associated predominantly with *Klebsiella pneumoniae* carbapenemase (KPC)-producing Enterobacterales, and the outbreak burden increased rapidly during the early 2020s COVID-19 pandemic (7, 8). KPC, classified as an Ambler class A β-lactamase, was initially reported in 2001 from clinical isolates in the United States and has since become the most prevalent carbapenemase globally (4, 6, 9). Due to its ability to break down all types of β-lactam (BL) rings and its resistance to classic β-lactamase inhibitors (BLIs) such as clavulanate, sulbactam, and tazobactam, conventional BLs or BL/BLIs are ineffective against KPC-producing Enterobacterales (10–12).

Recently, BL/BLI agents containing new KPC-active BLIs, including ceftazidime-avibactam, imipenem-relebactam, and meropenem-vaborbactam, have been developed and are being considered the treatment of choice against KPC-producing Enterobacterales (10–12). Among these agents, ceftazidime-avibactam was approved by the Korea Ministry of Food and Drug Safety on 22 December 2022, for the treatment of complicated intraabdominal infection, complicated urinary tract infection, and hospital-acquired pneumonia, and it was introduced in hospitals in October 2023 (13). However, recent data on antimicrobial activity of ceftazidime-avibactam against KPC-producing Enterobacterales are limited (14, 15). This is particularly important for three reasons. First, as the treatment cost of ceftazidime-avibactam is high, it is likely to be administered only to KPC-identified infections. Second, resistance to KPC-producing Enterobacterales may exist, caused by mechanisms other than carbapenemase, such as efflux pump or porin mutation (6). Third, antibiotics susceptibility test cards of automated systems for new BL/BLIs have not been widely adopted yet, and they require time for validation before use (16). For these reasons, we evaluated the antimicrobial activity of ceftazidime-avibactam against 188 isolates of KPC-producing Enterobacterales in comparison with other new US-FDA approved BL/BLIs, namely, imipenem-relebactam and meropenem-vaborbactam. For further investigation of the inhibitory activities of these new BLIs against non-susceptible strains, we conducted a cross-activity test using nine combinations of the individual BLs and BLIs in addition to a dose-escalation titration test of the new BLIs to determine whether susceptibility was dose-dependent for BLIs.

## MATERIALS AND METHODS

### Clinical and microbiological data

During the study period from January 2020 to June 2023, KPC-producing Enterobacterales isolated from a 2,000-bed tertiary care hospital were collected. In our hospital, clinical isolates of CRE obtained through either a CRE rectal screening test or a routine culture of clinical specimens underwent a multiplex real-time polymerase chain reaction (real-time PCR) to detect carbapenemase genes, including KPC, New Delhi metallo-β-lactamase (NDM), Verona integron-encoded metallo-β-lactamase (VIM), imipenemase-1 (IMP-1), and oxacillinase-48 (OXA-48). When carbapenemase genes were detected, the CPE isolates were sent to the Seoul Health and Environment Research Institute to confirm the subtypes of carbapenemase genes. To screen for carbapenemase genes, primer sequences categorized by the subtype were used (Table S5) (17–21). Among the CPE isolates, KPC-producing Enterobacterales were collected, and the medical records of the patients carrying those isolates were retrospectively reviewed. Only one isolate per patient was counted, with exceptions for duplicates if different species of KPC-producing Enterobacterales or the same species with a different antimicrobial susceptibility profile reported by the VITEK 2 automated system (bioMérieux, Marcy-l’Étoile, France) were isolated from the same patient. Brief clinical information was collected including age, sex, underlying disease, presence of clinical infection, definitive antibiotic treatment, and outcome. Clinical infection was defined as a bloodstream infection or organ infection accompanying symptoms or signs of infection requiring definitive antibiotic treatment according to the attending physicians’ discretion such as fever, leukocytosis, and elevated C-reactive protein levels. In-hospital mortality was evaluated as an outcome measure, with attributable mortality defined as death caused by uncontrolled infection due to KPC-producing Enterobacterales. This study was approved by the Institutional Review Board with the waiver of consent due to its retrospective nature (#2024-01-029).

### Antimicrobial susceptibility test

All collected KPC-producing Enterobacterales underwent antimicrobial susceptibility testing (AST) using the broth microdilution (BMD) method in accordance with the 2023 Clinical and Laboratory Standard Institute (CLSI) guideline for new BL/BLIs, including ceftazidime-avibactam, imipenem-relebactam, and meropenem-vaborbactam (22). The clinical breakpoints (CBPs) for the new BL/BLIs were as follows: ceftazidime-avibactam, minimum inhibitory concentration (MIC) ≤ 8/4 µg/mL was considered as susceptible, while MIC ≥ 16/4 µg/mL was classified as resistant; imipenem-relebactam, MIC ≤ 1/4 µg/mL was classified as susceptible, while MIC ≥ 4/4 µg/mL was classified as resistant; meropenem-vaborbactam, MIC ≤ 4/8 µg/mL was classified as susceptible, while MIC ≥ 16/4 µg/mL was classified as resistant. An MIC measured between the range of susceptible and resistant was defined as intermediate. Because the AST-N224 card of the VITEK 2 system does not report susceptibility results for colistin, it was tested additionally using the BMD method. For other antibiotics, the MIC results reported by the VITEK 2 system were utilized and interpreted in accordance with the 2023 CLSI guideline (22).

For a more in-depth investigation into the inhibitory activities of new BLIs, we conducted two additional experiments for the isolates that were non-susceptible to any of the three new BL/BLIs. First, cross-inhibition tests were performed using nine combinations of BL and new BLIs: ceftazidime-avibactam, ceftazidime-relebactam, ceftazidime-vaborbactam, imipenem-avibactam, imipenem-relebactam, imipenem-vaborbactam, meropenem-avibactam, meropenem-relebactam, and meropenem-vaborbactam. Second, despite the CLSI guideline recommending the use of a fixed concentration of BLIs for testing of BL/BLI agents, we conducted a dose-escalation titration test by increasing the concentration of new BLIs by two- and fourfold compared to the standard dose to investigate the therapeutic potential of new BL/BLI agents in the presence of higher BLI concentrations.

### Statistical analysis

Clinical characteristics were presented using descriptive statistics. To compare the MICs of BLs based on changes in BLIs, Log_2_ values of MIC were compared using Mann-Whitney *U* tests. All *P* values were two-tailed, and those <0.05 were considered statistically significant. GraphPad Prism software version 10 (GraphPad Software, San Diego, CA, USA) was used for all statistical analyses.

## RESULTS

### Baseline characteristic and clinical features

During the study period, 188 isolates of KPC-producing Enterobacterales were collected. The most common species was *Klebsiella* spp. (*n* = 138; 73.4%), followed by *Escherichia coli* (*n* = 35; 18.6%). The baseline characteristics of patients harboring KPC-producing Enterobacterales are presented in Tables S1 and S2. The mean age of the patients was 66.2 years, and males constituted 56.4%. Most patients (95.7%) had underlying diseases, with the most common being malignancy (62.2%), followed by diabetes mellitus (27.8%). Among the total isolates, 61.7% were asymptomatic colonizers, most commonly detected through rectal swab culture (74.1%), followed by urine culture (11.2%). Intra-abdominal infection was the most common type of clinical infections (50.0%), followed by pneumonia (27.8%) and urinary tract infection (11.1%). Patients with clinical infections were treated with colistin (60.9%) or aminoglycosides (46.4%), and the attributable mortality rate was 47.2%.

### Activity of new BL/BLIs against KPC-producing Enterobacterales

In the multiplex real-time PCR for carbapenemase genes and the subtype confirmation test, 186 isolates were found to carry the KPC-2 gene exclusively, while 2 isolates co-harbored NDM-1. Antibiotics activities were analyzed separately based on the carbapenemase-harboring status. Antibiotics activities against 186 KPC-2-producing Enterobacterales are presented in Table 1. Only two isolates were found to be resistant (1.1%), and the other 184 isolates were all susceptible (98.9%) to ceftazidime-avibactam. For susceptibility to imipenem-relebactam, 7 isolates (3.8%) were resistant, 6 isolates (3.2%) were intermediate, and 173 isolates (93.0%) were susceptible. A single isolate each (0.5%) was resistant and intermediate to meropenem-vaborbactam, whereas 184 isolates (98.9%) were susceptible. The resistance rate to colistin was 22.6%, and 77.4% were classified as intermediate according to the 2023 CLSI guideline. Based on the report of VITEK 2, most other BL agents were not active against KPC-2-producing Enterobacterales, while amikacin exhibited the highest susceptibility rate (82.8%), followed by gentamicin (82.8%), tigecycline (26.3%), and trimethoprim/sulfamethoxazole (25.8%). Two isolates co-harboring KPC-2 and NDM-1 were isolated from the same patient and were resistant to all new BL/BLIs, including ceftazidime-avibactam, imipenem-relebactam, and meropenem-vaborbactam (Table S3).

**TABLE 1:** Antibiotics activity against 186 KPC-2-producing enterobacterales^*a*^^,^^*e*^

Test method/antibiotics	MIC_50_ (μg/mL)	MIC_90_ (μg/mL)	Number of isolate (*n*, %)		
			S	I^*b*^	R
BMD					
Ceftazidime-avibactam	2/4	4/4	184 (98.9)	–	2 (1.1)
Imipenem-relebactam	0.25/4	1/4	173 (93.0)	6 (3.2)	7 (3.8)
Meropenem-vaborbactam	≤0.06/8	0.5/8	184 (98.9)	1 (0.5)	1 (0.5)
Colistin	1	16	–	144 (77.6)^*c*^	42 (22.6)
VITEK 2					
Ampicillin	≥32	≥32	0	1 (0.5)	185 (99.5)
Amoxicillin/clavulanate	≥32/16	≥32/16	0	2 (1.1)	184 (98.9)
Piperacillin/tazobactam	≥128/4	≥128/4	1 (0.5)	0	185 (99.5)
Aztreonam	≥64	≥64	1 (0.5)	0	185 (99.5)
Cefazolin	≥64	≥64	0	0	186 (100)
Cefoxitin	≥64	≥64	22 (11.8)	18 (9.7)	146 (78.5)
Cefotaxime	≥64	≥64	0	0	186 (100)
Ceftazidime	≥64	≥64	15 (8.1)	1 (0.5)	170 (91.4)
Cefepime	≥64	≥64	44 (23.7)	5 (2.7)	137 (73.7)
Ertapenem	≥8	≥8	2 (1.1)	0	184 (98.9)
Imipenem	≥16	≥16	2 (1.1)	6 (3.23)	178 (95.7)
Gentamicin	≥16	≥16	87 (46.8)	1.1	99 (53.2)
Amikacin	2	16	181 (97.3)	5.4	4 (2.2)
Ciprofloxacin	≥4	≥4	22 (11.8)	7 (3.8)	157 (84.4)
Trimethoprim/sulfamethoxazole	≥32/608	≥32/608	48 (25.8)	–	138 (74.2)
Tigecycline^*d*^	4	≥8	48 (25.8)	–	138 (74.2)

^*a*^ Clinical isolates co-harboring additional carbapenemase genes other than KPC-2 are presented separately in Table S2.

^*b*^ For piperacillin/tazobactam and cefepime, this range corresponds to SDD.

^*c*^ CLSI guideline does not provide CBP susceptible range for colistin, as blood concentration may not reach therapeutic range.

^*d*^ As CLSI does not provide CBP for Tigecycline, interpretation criteria of EUCAST were utilized.

^*e*^ Abbreviations: BL, β-lactam; BLI, β-lactamase inhibitor; KPC, *Klebsiella pneumoniae* carbapenemase; MIC, minimum inhibitory concentration; CLSI, Clinical and Laboratory Standards Institute; S, susceptible; I, intermediate; R, resistant; BMD, broth microdilution; SDD, susceptible-dose dependent; CBP, clinical breakpoint, EUCAST, European Committee on Antimicrobial Susceptibility Testing.

### Investigations for the inhibitory activities of new BLIs: cross-activity and dose-escalation tests

**Fig 1:**
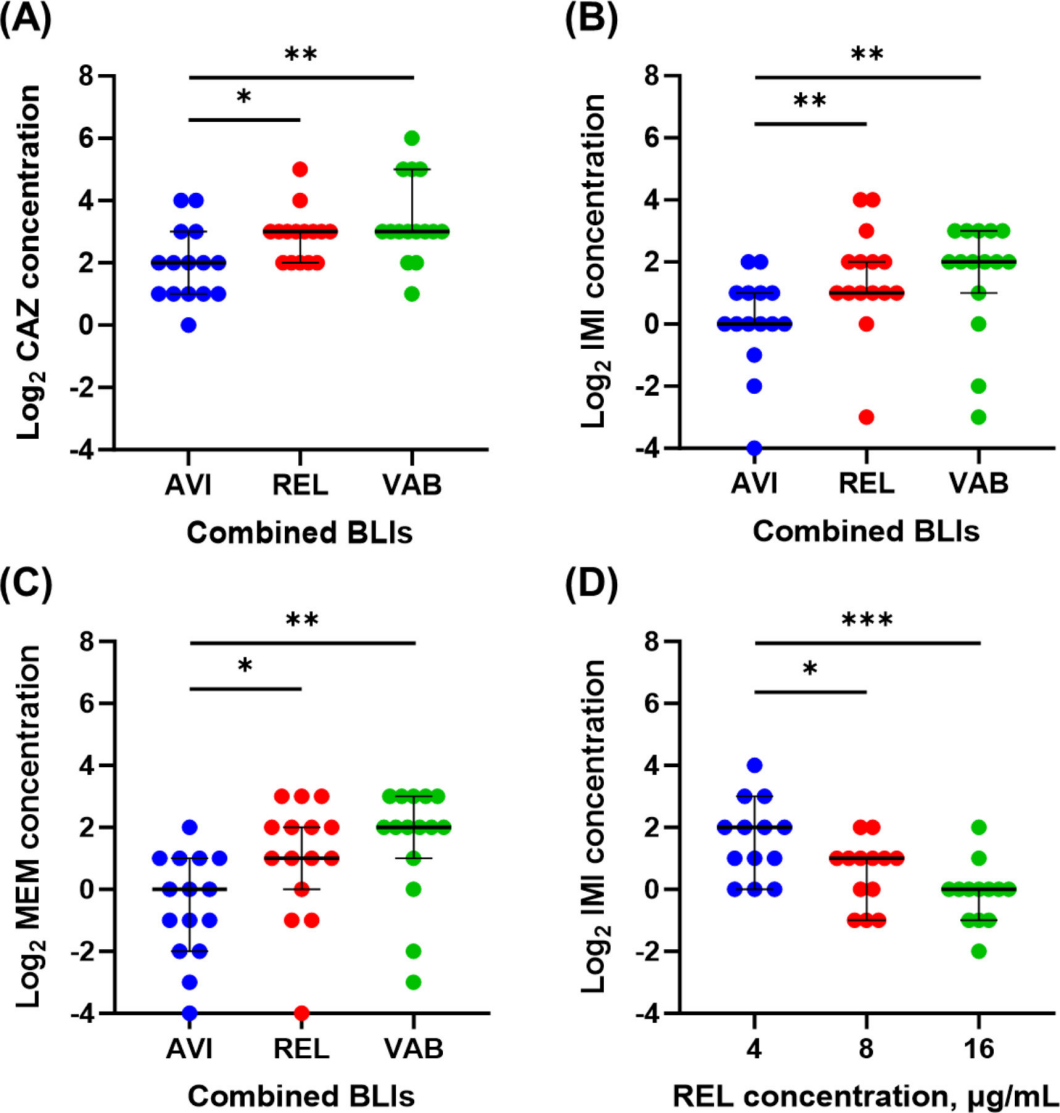
Cross-combination of BLs and new BLIs, and BLI titration tests. For further evaluation of activity of new BLIs against KPC-producing Enterobacterales, cross-combination tests of BLs and new BLIs were conducted. Non-susceptible strains for either CAZ-AVI, IMI-REL, or MEM-VAB were tested with (**A**) CAZ, (**B**) IMI, and (**C**) MEM in combination with AVI, REL, and VAB, respectively. As most strains in these tests were non-susceptible to IMI-REL, dose-escalation titration tests for REL in combination with IMI were statistically compared (**D**). Symbols of statistical comparison represent the following: *P ≤ 0.05; **P ≤ 0.01; and ***P ≤ 0.001. Abbreviations: BL, β-lactam; BLI, β-lactamase inhibitor; KPC, *Klebsiella pneumoniae* carbapenemase; CAZ, ceftazidime; IMI, imipenem; MEM, meropenem; AVI, avibactam; REL, relebactam; VAB, vaborbactam.

Based on the BMD tests, 15 KPC-2-producing Enterobacterales were non-susceptible to new BL/BLIs. Since susceptibility reports varied among the three new BL/BLIs, a cross-activity test with nine combinations of BLs and new BLIs was conducted to identify which component would not be active against these isolates (Table 2). In all cases resistant to ceftazidime-avibactam, susceptibility was not restored when avibactam was replaced with relebactam or vaborbactam; instead, the MIC of ceftazidime increased after the replacement. On the other hand, among isolates non-susceptible to imipenem-relebactam or meropenem-vaborbactam, 7 of 11 (63.6%) imipenem-relebactam non-susceptible isolates and both (100.0%) of the meropenem-vaborbactam non-susceptible isolates became susceptible when avibactam replaced the new BLIs. To quantitatively compare the inhibitory activity of new BLIs, the MICs of BLs were compared using log_2_ scales in each case of cross-combination of BLs and BLIs (Fig. 1A through C). Regardless of the type of BLs, combinations with avibactam showed statistically significant efficacy in lowering MICs compared to relebactam and vaborbactam (all *P* < 0.05).

**TABLE 2:** Activity of nine cross-combination of BLs and new BLIs against 15 KPC-2-producing Enterobacterales non-susceptible to new BL/BLIs^*a*^

Non-susceptible new BL/BLI	Species	Ceftazidime			Imipenem			Meropenem		
		Avibactam	Relebactam	Vaborbactam	Avibactam	Relebactam	Vaborbactam	Avibactam	Relebactam	Vaborbactam
Ceftazidime-avibactam	*E. coli*	16, R	32, R	64, R	≤0.06, S	0.12, S	0.12, S	≤0.06, S	≤0.06, S	≤0.06, S
	*K. pneumoniae*	16, R	16, R	32, R	1, S	1, S	1, S	0.25, S	0.5, S	0.5, S
Imipenem-relebactam	*K. pneumoniae*	2, S	4, S	8, S	1, S	4, R	4, R	0.5, S	2, S	4, S
	*K. pneumoniae*	8, S	8, S	4, S	0.5, S	2, I	4, R	0.25, S	1, S	1, S
	*K. pneumoniae*	4, S	8, S	8, S	2, I	4, R	4, R	2, S	4, S	2, S
	*K. pneumoniae*	2, S	8, S	8, S	2, I	4, R	8, R	2, S	4, S	2, S
	*K. pneumoniae*	8, S	8, S	32, R	2, I	8, R	8, R	1, S	4, S	2, S
	*S. marcescens*	1, S	4, S	4, S	1, S	2, I	2, I	2, S	8, I	4, S
	*K. pneumoniae*	2, S	4, S	8, S	1, S	2, I	4, R	0.5, S	2, S	2, S
	*K. pneumoniae*	4, S	8, S	8, S	0.25, S	2, I	0.25, S	0.12, S	0.5, S	0.12, S
	*K. pneumoniae*	2, S	4, S	8, S	1, S	2, I	4, R	0.5, S	2, S	1, S
	*K. pneumoniae*	4, S	8, S	32, R	1, S	2, I	4, R	1, S	2, S	2, S
	*K. pneumoniae*	2, S	8, S	8, S	4, R	16, R	8, R	2, S	8, I	4, S
Imipenem-relebactam and Meropenem-vaborbactam	*K. pneumoniae*	4, S	4, S	2, S	2, I	4, R	8, R	4, S	4, S	8, I
	*K. pneumoniae*	4, S	8, S	8, S	4, R	16, R	8, R	1, S	8, I	16, R

^*a*^ Abbreviations: BL, β-lactam; BLI, β-lactamase inhibitor; KPC, *Klebsiella pneumoniae* carbapenemase; S, susceptible; I, intermediate; R, resistant.

Next, to determine whether the insufficient activity of new BLIs results from a low concentration, additional BMD tests were conducted by escalating the dose of new BLIs (Table 3). Increasing the dose of all new BLIs increased susceptibility to BLs. Since most strains in these tests were non-susceptible to imipenem-relebactam, a quantitative comparison of the log_2_ MIC of imipenem according to relebactam concentration was conducted (Fig. 1D). The log_2_ MIC of imipenem decreased significantly with increasing concentrations of relebactam (both *P* < 0.05).

**TABLE 3:** Dose-escalations BLI titrations against 15 KPC-2-producing Enterobacterales non-susceptible to new BL/BLIs^*b*^

Non-susceptible new BL/BLI	Species	Ceftazidime			Imipenem			Meropenem		
		Avibactam 4 µg/mL	Avibactam 8 µg/mL	Avibactam 16 µg/mL	Relebactam 4 µg/mL	Relebactam 8 µg/mL	Relebactam 16 µg/mL	Vaborbactam 8 µg/mL	Vaborbactam 16 µg/mL	Vaborbactam 32 µg/mL
Ceftazidime-avibactam	*E. coli*	16, R	2, S	0.25, S						
	*K. pneumoniae*	16, R	4, S	1, S						
Imipenem-relebactam	*K. pneumoniae*	2, S			2, I	2, I	1, S			
	*K. pneumoniae^a^*	8, S			1, S	0.5, S	0.5, S			
	*K. pneumoniae*	4, S			4, R	2, I	2, I			
	*K. pneumoniae*	2, S			4, R	2, I	1, S			
	*K. pneumoniae*	8, S			4, R	2, I	1, S			
	*S. marcescens*	1, S			1, S	0.5, S	0.5, S			
	*K. pneumoniae*	2, S			4, R	2, I	1, S			
	*K. pneumoniae^a^*	4, S			1, S	0.5, S	0.25, S			
	*K. pneumoniae*	2, S			2, I	1, S	0.5, S			
	*K. pneumoniae*	4, S			2, I	1, S	1, S			
	*K. pneumoniae*	2, S			8, R	4, R	4, R			
Imipenem-relebactam and Meropenem-vaborbactam	*K. pneumoniae^a^*	4, S			8, R	2, I	1, S	4, S	1, S	0.5, S
	*K. pneumoniae*	4, S			16, R	4, R	1, S	8, R	1, S	0.5, S

^*a*^ Three isolates, previously reported as non-susceptible to new BL/BLIs, were found to be susceptible in this dose-escalation test. The difference in MIC was within a twofold range, which is considered acceptable variation between runs.

^*b*^ Abbreviations: BL, β-lactam; BLI, β-lactamase inhibitor; KPC, *Klebsiella pneumoniae* carbapenemase; S, susceptible; I, intermediate; R, resistant.

Last, to compare the inhibitory activity of avibactam and relebactam at higher concentrations, MICs of imipenem were evaluated at concentrations of 8 and 16 µg/mL of avibactam and relebactam, respectively (Table S4). Overall, the avibactam combination exhibited better susceptibility at high concentrations, and the log_2_ MIC of imipenem was significantly lower with avibactam than with relebactam at concentrations of 8 and 16 µg/mL, respectively (both *P* < 0.05).

## DISCUSSION

The present study was conducted using 188 KPC-producing Enterobacterales collected over a period of three and a half years, from January 2020 to June 2023, reflecting the most recent clinical isolates. Not only were colonizers acquired from CRE screening tests evaluated, but isolates causing clinically significant infections accounted for 38.3% of the total isolates, with an attributable mortality rate of 47.2%. Among the total 188 isolates of KPC-producing Enterobacterales, 117 isolates were obtained through rectal screening, and out of these, 31 isolates (31/117, 26.5%) were associated with clinical infections. Also, among the 116 isolates of asymptomatic colonizers, 86 isolates were obtained through rectal screening (86/116, 74.1%). These results indicate that isolates obtained through rectal screening have a higher proportion of asymptomatic colonizers compared to clinical infections. Colistin and aminoglycosides, both known for their potential nephrotoxicity, constituted the sole therapeutic modalities prior to the introduction of new BL/BLIs. This implies that KPC-producing Enterobacterales pose a substantial clinical burden on domestic health, consistent with previous reports (7, 8, 23). Notably, ceftazidime-avibactam exhibited excellent activity against 186 isolates exclusively carrying KPC-2 as a carbapenemase, with only two isolates (1.1%) demonstrating resistance. While NDM-1 co-harboring isolates were uniformly resistant to ceftazidime-avibactam, these comprised only 2 of 188 isolates (1.1%). As the presence of metallo-β-lactamase co-existing with KPC can be identified easily through multiplex carbapenemase gene real-time PCR, ceftazidime-avibactam could be a reliable treatment of choice for KPC-producing Enterobacterales.

Interestingly, both KPC-2-producing isolates resistant to ceftazidime-avibactam became susceptible to ceftazidime when the concentration of combined avibactam was increased twofold (8 µg/mL). The recovery of BL susceptibility, depending on the concentration of BLI, was also observed in the dose-escalation titration test for imipenem-relebactam and meropenem-vaborbactam. A recent report revealed that, whereas high-level ceftazidime-avibactam resistance was associated with metallo-β-lactamase or a point mutation in the *bla*_KPC-2_ gene, low-level ceftazidime-avibactam resistance was associated with overexpression and increased copy number of wild-type *bla*_KPC-2_ (24). Therefore, it would be a reasonable inference that the increasing concentration of avibactam may overcome the inoculum effect of overexpressed *bla*_KPC-2_. The pharmacokinetic data suggest that *C*_max_ of ceftazidime and avibactam are 90.4 µg/mL and 14.6 µg/mL, and *T*_1/2_ are 2.76 hours and 2.71 hours, respectively (after multiple doses of ceftazidime 2 g and avibactam 0.5 g) (25). With this standard regimen, a blood concentration of avibactam greater than 8 µg/mL would be attained only for several hours. Since the time above the MIC is the most important factor for β-lactam antibiotics (26), further strategies to achieve a higher concentration of ceftazidime-avibactam, such as prolonged infusion or shortening infusion intervals, need to be developed for isolates with low-level resistance. It is already known that prolonged infusion of BLs reduced mortality and antibiotic-related adverse events were generally mild (27). According to recent retrospective case series of ceftazidime-avibactam administered through prolonged infusion, clinical cure and microbiological eradication were achieved at high levels. Neither antibiotic-related adverse events nor ceftazidime-avibactam resistance were noted during the follow-up period. Additionally, there was no instability of the ceftazidime-avibactam during the period of prolonged infusion (28).

In addition, we directly compared the activities of three new BLIs, by conducting cross-combination tests. Our results indicate that avibactam exhibited the most excellent activity against KPC-producing Enterobacterales among the three new BLIs. While some previous studies implied better activity of ceftazidime-avibactam compared to imipenem-relebactam or meropenem-vaborbactam, there were no direct comparisons between BLIs (29–31). On the other hand, both KPC-2-producing isolates resistant to ceftazidime-avibactam became susceptible to imipenem-avibactam and meropenem-avibactam combinations. When comparing BLs combined with avibactam, meropenem-avibactam (15/15, 100.0%) showed a better susceptibility profile than imipenem-avibactam (9/15, 60.0%) or ceftazidime-avibactam (13/15, 86.7%). These findings suggest that further investigations are warranted for BL combinations based on avibactam.

There are several limitations in the present study. First, it was a single-center study conducted over a relatively short period. Nevertheless, we evaluate the most recently collected KPC-producing Enterobacterales isolated, comprising the largest numbers to date, representing the susceptibility profile at the time of the global introduction of ceftazidime-avibactam. Second, we did not assess the copy numbers of KPC gene expression among non-susceptible isolates. However, by demonstrating that the increasing concentration of new BLIs restored susceptibility and lowered MIC of BLs, we could phenomenologically suggest that overexpression of the KPC gene is related to resistance. Last, although we suggest that increasing avibactam concentration would overcome low-level resistances to ceftazidime-avibactam, it was not proved clinically. Further clinical or animal studies that can support this *in vitro* finding are required.

In conclusion, ceftazidime-avibactam exhibited excellent activity against recently isolated KPC-2-producing Enterobacterales, except in those co-harboring metallo-β-lactamase. Cross-combination tests against non-susceptible isolates suggest that the inhibitory activity of avibactam was superior to those of relebactam or vaborbactam. Increasing the dose of new BLIs corresponded to the increased susceptibility to BLs, suggesting that a high-concentration regimen needs to be developed.

## SUPPLEMENTAL MATERIAL

Supplemental material spectrum.00344-24-s0001.docx Tables S1-S5.

ASM does not own the copyrights to Supplemental Material that may be linked to, or accessed through, an article. The authors have granted ASM a non-exclusive, world-wide license to publish the Supplemental Material files. Please contact the corresponding author directly for reuse.
