# Supplementary material for: Antimicrobial activity of ceftazidime-avibactam against KPC-2-producing Enterobacterales: a cross-combination and dose-escalation titration study with relebactam and vaborbactam
Source: Microbiol Spectr. 2024 Apr 30;12(6):e00344-24. doi: 10.1128/spectrum.00344-24 (PMC11237450; doi:10.1128/spectrum.00344-24)
Supplement: Supplemental material — Tables S1-S5. [file spectrum.00344-24-s0001.docx]

**Supplementary Table 1. Clinical characteristics of 188 infection/colonization events caused by KPC-producing Enterobacterales**

| **Variables** | **Total**  **N=188** | ***Klebsiella* spp.**  **N=138** | ***E. coli***  **N=35** | **Others^*^**  **N=15** |
| --- | --- | --- | --- | --- |
|  | **n/N (%)** | | | |
| **Demographic characteristics** |  |  |  |  |
| Age, year | 66.2 ± 13.8 | 66.4 ± 13.8 | 67.1 ± 12.4 | 62.3 ± 17.3 |
| Male sex | 106/188 (56.4) | 79/138 (57.2) | 17/35 (48.6) | 10/15 (66.7) |
| Underlying diseases | 180/188 (95.7) | 132/138 (95.7) | 33/35 (94.3) | 15/15 (100.0) |
| Diabetes mellitus | 50/180 (27.8) | 34/132 (25.8) | 11/33 (33.3) | 5/15 (33.3) |
| Lung disease | 25/180 (13.9) | 19/132 (14.4) | 5/33 (15.2) | 1/15 (6.7) |
| Cardiovascular disease | 35/180 (19.4) | 28/132 (21.2) | 6/33 (18.2) | 1/15 (6.7) |
| Liver disease | 33/180 (18.3) | 25/132 (18.9) | 7/33 (21.2) | 1/15 (6.7) |
| Renal disease | 30/180 (16.7) | 24/132 (18.2) | 3/33 (9.1) | 3/15 (20.0) |
| Malignancy | 112/180 (62.2) | 81/132 (61.4) | 21/33 (63.6) | 10/15 (66.7) |
| Cerebrovascular disease | 14/180 (7.8) | 13/132 (9.8) | 1/33 (3.0) | 0 /15 (0.0) |
| **Asymptomatic colonizer** | 116/188 (61.7) | 80/138 (58.0) | 24/35 (68.6) | 12/15 (80.0) |
| Rectal swab | 86/116 (74.1) | 60/80 (75.0) | 18/24 (75.0) | 8/12 (66.7) |
| Urine culture | 13/116 (11.2) | 10/80 (12.5) | 2/24 (8.3) | 1/12 (8.3) |
| Others | 17/116 (14.7) | 10/80 (12.5) | 4/24 (16.7) | 3/12 (25.0) |
| **Clinical infection** | 72/188 (38.3) | 58/138 (42.0) | 11/35 (31.4) | 3/15 (20.0) |
| Type of infection |  |  |  |  |
| Intra-abdominal infection | 36/72 (50.0) | 29/58 (50.0) | 4/11 (36.4) | 3/3 (100.0) |
| Pneumonia^†^ | 20/72 (27.8) | 19/58 (32.8) | 1/11 (9.1) | 0/3 (0.0) |
| Urinary tract infection | 8/72 (11.1) | 5/58 (8.6) | 3/11 (27.3) | 0/3 (0.0) |
| Catheter-related infection | 4/72 (5.6) | 3/58 (5.2) | 1/11 (9.1) | 0/3 (0.0) |
| Skin and soft tissue infection | 4/72 (5.6) | 2/58 (3.4) | 2/11 (18.2) | 0/3 (0.0) |
| Definitive antibiotics | 69/72 (95.8) | 55/58 (94.8) | 11/11 (100.0) | 3/3 (100.0) |
| Aminoglycoside | 32/69 (46.4) | 28/55 (50.9) | 3/11 (27.3) | 1/3 (33.3) |
| Colistin | 42/69 (60.9) | 32/55 (58.2) | 8/11 (72.7) | 2/3 (66.7) |
| In-hospital mortality |  |  |  |  |
| All-cause mortality | 41/72 (56.9) | 33/58 (56.9) | 5/11 (45.5) | 3/3 (100.0) |
| Attributable mortality | 34/72 (47.2) | 26/58 (44.8) | 5/11 (45.5) | 3/3 (100.0) |

Data are expressed as the number (%) of patients or mean ± SD. As separate infection/colonization events caused by different species or antibiograms were counted individually, the data represents 188 events in 138 patients. Demographic characteristics of 138 patients, evaluated without duplication, are presented as Supplementary Table 2. ^*^Others include 15 *Citrobacter* spp.,2 *Enterobacter cloacae* complex, and 1 *Serratia marcescens*. ^†^Including 2 case of lung abscess.

Abbreviations: KPC, *Klebsiella pneumoniae* carbapenemase; SD, standard deviation

**Supplementary Table 2. Demographic characteristics of 138 patients harboring KPC-producing Enterobacterales**

| **Variables** | **Total**  **N =138** |
| --- | --- |
| **Demographic characteristics** |  |
| Age, year | 66.91 ± 14.1 |
| Male sex | 62 (44.9) |
| Underlying diseases | 131 (94.9) |
| Diabetes mellitus | 38 (29.0) |
| Lung disease | 21 (16.0) |
| Cardiovascular disease | 29 (22.1) |
| Liver disease | 25 (19.1) |
| Renal disease | 22 (16.8) |
| Malignancy | 83 (63.4) |
| Cerebrovascular disease | 13 (9.9) |

Data are expressed as the number (%) of patients or mean ± SD.

Abbreviations: KPC, *Klebsiella pneumoniae* carbapenemase; SD, standard deviation

**Supplementary Table 3. Antibiotics activity against two KPC-2 and NDM-1 co-harboring Enterobacterales**

| **Carbapenemase genes** | **Species** | **BMD** | | | | **VITEK 2** | | | | | |
| --- | --- | --- | --- | --- | --- | --- | --- | --- | --- | --- | --- |
|  |  | Ceftazidime-avibactam | Imipenem-relebactam | Meropenem-vaborbactam | Colistin | Ampicillin | Amoxicillin-clavulanate | Piperacillin-tazobactam | Aztreonam | Cefazolin | Cefoxitin |
| KPC-2, NDM-1 | *E. cloacae* | ≥128, R | ≥128, R | 64, R | 0.5, I | ≥32, R | ≥32, R | ≥128, R | ≥64, R | ≥64, R | ≥64, R |
|  | *K. pneumoniae* | ≥128, R | 64, R | 64, R | 0.5, I | ≥32, R | ≥32, R | ≥128, R | ≥64, R | ≥64, R | ≥64, R |

*Continued below,*

| **Carbapenemase genes** | **Species** | **VITEK 2** | | | | | | | | | |
| --- | --- | --- | --- | --- | --- | --- | --- | --- | --- | --- | --- |
|  |  | Cefotaxime | Ceftazidime | Cefepime | Ertapenem | Imipenem | Gentamicin | Amikacin | Ciprofloxacin | TMP/SMX | Tigecycline |
| KPC-2, NDM-1 | *E. cloacae* | ≥64, R | ≥64, R | ≥64, R | ≥8, R | ≥16, R | ≤1, S | ≤2, S | ≥4, R | ≤20, S | ≥8, R |
|  | *K. pneumoniae* | ≥64, R | ≥64, R | ≥64, R | ≥8, R | ≥16, R | ≤1, S | ≤2, S | ≤0.25, S | ≤20, S | 1, R |

All these species were isolated from the same patient.

Abbreviations: KPC, *Klebsiella pneumoniae* carbapenemase; NDM, New Delhi metallo-β-lactamase; S, susceptible; I, intermediate; R, resistant; BMD, broth microdilution

**Supplementary Table 4. Dose-escalation titration tests of imipenem- avibactam against 8 strains non-susceptible to imipenem with a relebactam concentration of 8 μg/mL**

| **Non-susceptible new BL/BLI** | **Species** | **Imipenem** | | | |
| --- | --- | --- | --- | --- | --- |
|  |  | Avibactam  *8 μg/mL* | Avibactam  *16 μg/mL* | Relebactam  *8 μg/mL* | Relebactam  *16 μg/mL* |
| **Imipenem-relebactam**  *8 μg/mL* | *K. pneumoniae* | 1, S | 0.5, S | 2, I | 1, S |
|  | *K. pneumoniae* | 2, I | 1, S | 2, I | 2, I |
|  | *K. pneumoniae* | 1, S | 0.5, S | 2, I | 1, S |
|  | *K. pneumoniae* | 0.5, S | 0.5, S | 2, I | 1, S |
|  | *K. pneumoniae* | 0.5, S | 0.25, S | 2, I | 1, S |
|  | *K. pneumoniae* | 2, I | 2, I | 4, R | 4, R |
|  | *K. pneumoniae* | 0.5, S | 0.25, S | 2, I | 1, S |
|  | *K. pneumoniae* | 0.5, S | 0.5, S | 4, R | 1, S |

Abbreviations: BL, β-lactam; BLI, β-lactamase inhibitor; S, susceptible; I, intermediate; R, resistant

**Supplementary Table 5. Primer sequences and product sizes of the carbapenemase genes**

| Carbapenemase genes | Primer sequences (5'-3') | Product sizes (bp) |
| --- | --- | --- |
| *bla*KPC | F: ATGTCACTGTATCGCCGTCT  R: TTTTCAGAGCCTTACTGCCC | 893 |
| *bla*NDM | F: ATGGAATTGCCCAATATTATGCAC  R: TCAGCGCAGCTTGTCGGC | 612 |
| OXA-48 | F: TTGGTGGCATCGATTATCGG  R: GAGCACTTCTTTTGTGATGGC | 744 |
| IMP | F: GTTTATGTTCATACWTCG  R: GGTTTAAYAAAACAACCAC | 432 |
| VIM | F: TGGTCTACATGACCGCGTCT  R: CGACTGAGCGATTTGTGTG | 766 |

Note: F, Forward primer; R, Reverse primer; KPC, Klebsiella pneumoniae carbapenemase; NDM, New Delhi metallo-β-lactamase; OXA-48, Oxacillin hydrolyzing enzymes-48; IMP, imipenemase metallo-β-lactamase; VIM, Verona integron-mediated metallo-β-lactamase; bp, base pairs.
